# Cervical cancer screening practice and associated factors among women employees in Wolaita Zone hospitals, Southern Ethiopia, 2017: cross-sectional study

**DOI:** 10.11604/pamj.2022.42.318.34675

**Published:** 2022-08-29

**Authors:** Getachew Tesfaye, Shifera Yedenekal, Million Abera, Mihretu Lakew, Tilahun Wodaynew, Abebe Mamo

**Affiliations:** 1 Wolaita Zone Health Office, Wolaita, Ethiopia,; 2 Department of Health Behavior and Society, Jimma University, Jimma, Ethiopia,; 3 School of Nursing, Jimma University, Jimma, Ethiopia,; 4 Jimma Zone Health Office, Jimma, Ethiopia

**Keywords:** Cervical cancer, screening practice, woman employees, Wolaita Zone hospital

## Abstract

**Introduction:**

cervical cancer is a global public health problem affecting women worldwide. There is very low participation rate in screening practice for cervical cancer in low-resource countries like Ethiopia. So the aims of this study is to assess cervical cancer screening practice and associated factors among women employees in Wolaita Zone hospitals, Southern Ethiopia.

**Methods:**

facility based cross-sectional study design was conducted from March 1-April 30, 2017. Simple random sampling technique was employed to select 401 study participants. Pre-tested self-administered questionnaire was used. Logistic regression was performed to assess association between dependent and independent variables with 95% confidence interval (CI) and p-value less than 0.05 was set to declare association.

**Results:**

about 120 (30.5%) participants were screened for cervical cancer. Age, source of information from health professions, being adherence supporter, sex with more than one partner, sexual transmitted infection, increase in attitude and knowledge score were significant predictors of cervical cancer screening practice.

**Conclusion:**

magnitude of cervical cancer screening practice among age eligible women is still low. Age, being adherence supporter, source of information from health care professionals, history of multiple sexual p artners, sexually transmitted infection, knowledge and attitude were important predictors of cervical cancer screening practice. Hospitals in collaboration with town administration should put priority on cervical cancer prevention by establishing cervical cancer screening campaign.

## Introduction

Cervical cancer is a global public health problem accounting for the fourth most common cancer-affecting women worldwide and in developing countries, it is the third leading cause of cancer death [1,2]. The world has estimated population of 527,624 women aged 15 years and older who are diagnosed with cervical cancer and 265,672 die from the disease annually worldwide, 87% occurring in sub-Sahara countries [3]. In Ethiopia, there are 27.19 million women aged 15 years and older who are at risk of developing cervical cancer, and every year 7,095 women diagnosed with cervical cancer and 4,732 die from the disease [4] and evidences suggested that more than 99% of cervical cancer cases are linked to Human Papilloma-Virus (HPV) [5,6]. According to study conducted in Botswana and Uganda, the cervical cancer screening rate of the study participants were 40.0% and 19% respectively [7,8]. The disparity in cervical cancer diagnosis and subsequent mortality between high- and low-resource countries is due largely to the low rate of screening for pre-invasive cervical disease and limited treatment options in low resource settings. Even if, the ministry of health has been trying to deal with this problem by providing resources so as to reduce the incidence of cervical cancer, in Ethiopia, only 1% of age eligible women receive effective screening for cervical cancer and 90% of women have never had a pelvic examination [9,10].

The majority of cervical cancer deaths occur in women who never screened and in women with well-described sexual and reproductive risk factors, such as an early sexual debut, a history of multiple sexual partners, and a high number of live births [11]. Low level of awareness, lack of effective screening programs, overshadowed by other health priorities (such as AIDS, TB, malaria) and insufficient attention to women´s health are the possible factors for the observed higher incidence rate of cervical cancer in the country [9,12]. Previous studies conducted in the country tried to assess level of knowledge of study participants on its risk factors, sign, symptoms, and prevention methods, the frequency of positive and negative attitude towards cervical cancer [13,14]. But little is known about cervical cancer screening practice and associated factors till this research is done. Besides this, there is dearth of evidence concerning cervical cancer screening practice and associated factors in the study area. Therefore, the main purpose of this research is to identify factors associated with cervical cancer screening practice and to recommend ways and set directions to increase cervical cancer screening and the study participants get an insight about cervical cancer and screening during the data collection period and long term benefit from study finding.

## Methods

**Study area and study period:** the study was conducted in Wolaita Zone, Southern Ethiopia. Wolaita Zone is located at a distance of 153Km from capital of SNNPR (Hawassa) and 328Km south of the capital city (Addis Ababa). Wolaita Zone is administratively divided into twelve districts and three town administration. The Zone has five hospitals, 71 health centers, 372 health posts and 98 private clinics. Based on the projection of 2007 population and housing census the population of Wolaita Zone is about 1,888,390 in 2014. Currently there are three hospitals providing cervical cancer screening and treatment, Otona, Christian and Dubo. The study was conducted in these three hospitals from March 1-April 30, 2017.

**Study design:** facility based cross-sectional study design was employed.

**Population:** all women employees in the hospitals (Otona, Christian and Dubo) with the age range of 25-49 years [15] were the source of population and all selected women employee in those hospitals with the age range of 25-49 years were the study population during the data collection period. Those who were critically sick during the data collection period excluded from the study.

**Sample size determination and sampling procedure:** the sample size was determined using a single population proportion formula considering the following assumptions: 19.8% proportion of women who underwent cervical cancer screening [12], 95% confidence interval and 3% margin of error. Single proportion formula:


$$n=\frac{{\left(Z_{a/2}\right)}^{2}p(1-P)}{d^{2}}=\frac{{\left(1.96\right)}^{2}\times 0.198\times 0.802}{{\left(0.03\right)}^{2}}=677$$

(Z)^2^ at 95% confidence interval = (1.96)^2^; d = marginal of error = 3% = 0.03; n = sample size P = estimated proportion (19.8%). Since the total number of study population is < 10,000 (N=880) using the correction formula:


$$nf=\frac{n}{1+\frac{\left(n\right)}{N}}=\frac{677}{1+\frac{\left(677\right)}{880}}=382$$

Where, nf is the final sample size, n is the calculated sample size which is 677, N is the total number of woman employees in the three study hospitals (880). The final sample size by adding non response rate 5% (382x0.05 + 382) was 401. The health facilities where routine cervical cancer screening and treatment by using visual inspection with acetic acid and providing the service for all women were included. The health facilities were; Otona hospital (460), Christian hospital (180) and Dubo hospital (240) with a total of 880 female employees. Then the total sample size was allocated for each hospitals using probability proportionate to their size and then the allocated sample size from Otona hospital, Christian hospital and Dubo hospital were 210, 82 and 109 respectively. Finally, simple random sampling technique was employed to select study participants from each respective Hospital.

### Study variables

**Dependent variables:** cervical cancer screening practice.

**Independent variables:** socio-demographic characteristics: (age, marital status, educational status, religion, income, occupation), knowledge towards cervical cancer screening practice, attitude towards cervical cancer screening practice, sources of information´s, life style and behavioral factors.

**Data collection tools:** a pre-tested semi-structured self-administered questionnaire was used and facilitated by four female nurses and supervised by two health officers. Questionnaire was adapted from similar studies and it had five parts. The first part has socio-demographic characteristics, the second, the third, fourth and fifth parts are knowledge, attitude, practice on cervical cancer screening and their life style and behavioral factors respectively.

**Data quality control:** pre-testing on 5% of a sample drawn from Arbaminch hospital was conducted prior to data collection to assess the cultural sensitivity and clarity of the items in the questionnaire. As already mentioned semi- structured questionnaire in English was translated into Amharic language and back translated into English by another person to check its validity. Clarification was provided to participants prior to distribution of the paper. Four data collectors and two supervisors were trained before the actual data collection period regarding the approach, objective of the study and ethical issue. The entered data was checked for completeness at the beginning and middle stage of the work. Data cleaning was conducted at the end of the data entry. Further, confirmatory principal component analysis was performed to validate and check whether items were loaded to their respective constructs. Factor loading score of ≥40% and varimax method of rotation was considered to load items. It showed that the items measuring attitude towards cervical cancer. Here, Eigen value of >1 was considered for construct validity.

**Data processing and analysis:** after the data collection, data was checked manually for its completeness. The data was entered and coded by using EPI-Data 3.1 and after its completion; it was exported to SPSS Version 21 for further analysis. Variables reached a p-value of 0.25 on bivariate analysis were included in multiple logistic regression analysis. Multivariate analysis using backward stepwise selection method was employed and p-values of less than 0.05 were taken to represent significance. The degree of association between the independent and dependent variables was analyzed using odds ratios with 95% confidence intervals. Statistical significance was declared at P-value < 0.05.

**Ethical consideration:** ethical clearance was obtained from research ethical committee of school of public health in Jimma University. Following this, SNNPR health bureau and Wolaita Zonal health department were informed on study objective and study permission was obtained. Then a written consent was secured from the study participants through informed consent. The participants assured that the information they were going to give was used only for the purpose of the study and confidentiality was kept. Though having conversation about cervical cancer might cause anxiety. But to handle such conditions the data collectors assured the participants well about the objective of the study.

### Operational definitions

**Knowledge about cervical cancer screening:** was assessed by using items with yes or no response format about sign and symptoms, risk factors, method of prevention, frequency of screening, procedure of screening and eligibility for screening. Totally knowledge was assessed by nineteen items. Finally measures for knowledge about cervical cancer was scored and knowledge of study participants analyzed as continuous variable [16].

**Attitude assessment:** attitude was assessed by six questions put on Likert´s scale. The questions on Likert´s scale had positive and negative responses that ranged from strongly agree, agree, neither agree nor disagree, disagree and strongly disagree. The scoring system used with respects to respondents´ responses as follows: strongly agree scored 5, agree 4, neither agree nor disagree 3, disagree 2, strongly disagree 1. The responses were summed up and a total score computed for each respondent. Finally attitude of study participants analyzed as continuous variable.

**Practice:** to assess practice, question was delivered in yes or no option to assess the past five years respondent´s action towards screening and those who ever screened once or more within the past five years regarded as having screening practice and those who never screened was regarded as having no practice on screening [4].

## Results

**Socio-demographic characteristics:** a total of 393 women employees participated in this study with (98%) response rate. The tables showed that from 393 total respondents, 203 (51.6%) were in the age range 25-29 years. Majority of the respondents, 270 (68.7%) were married (Table 1).

**Table 1:** percentage distribution of the woman employees by selected socio-demographic characteristics, in Wolaita Zone hospitals, Ethiopia, June 2017 (n=393)

Variables	Categories	Frequency (n)	%
**Age in (years)**	25-29	203	51.6
	30-34	109	27.7
	35-39	44	11.2
	40-44	28	7.2
	45-49	9	2.3
**Marital status**	Married	270	68.7
	Single	123	31.3
**Religion**	Protestant	237	60.3
	Orthodox	97	24.7
	catholic	50	12.7
	Muslim	9	2.3
**Ethnicity**	Wolaita	303	77.1
	Gamo	28	7.1
	Kembata	27	6.9
	Gurage	20	5.1
	Others	15	3.8
**Level of education**	Below certificate	42	10.7
	Certificate	43	10.9
	Diploma	121	30.8
	First degree and plus	187	47.6
**Occupation**	Health care professionals	172	43.8
	Administrative staff	71	18.1
	Adherence supporter	24	6.0
	Cleaners	126	32.1

**Practice of cervical cancer screening:** among all the study participants, 120 (30.5%) screened for cervical cancer. Among these 98 (81.7%) were screened by self-initiation and the rest 22 (5.6%) were due to health care professionals request. Most of the study participants who had screened for cervical cancer, 112 (93.3) were screened once and the rest 8 (6.7%) screened more than once.

**Reason mentioned for not to screen cervical cancer:** the majority of respondents never had cervical cancer screening. Respondents who have no screening practice were asked for their reasons for not to screen and among those who heard of screening ninety five mentioned as they were not decided well, 80 mentioned as they were afraid of the screening result.

**Source of information for cervical cancer screening:** about 393(98%) of the study participants heard about cervical cancer but the source from which they heard was different. Most of them heard from health professionals (44%) and secondly from radio/television (33%).

**Life style and sexual behavior factors:** risk exposure of participants had assessed and out of all the study participants, 135 (34.3%) had used modern contraceptives. Of those who used modern contraceptives 75 (55.5%) used oral contraceptive for greater than five years. Over all participants, 21 (5.3%), had sex before the age of fifteen years. Some of the study participants had sexually transmitted infection 94 (23.9%).

**Predictors of cervical cancer screening practice:** in multivariate analysis age of the women, adherence supporter, and source of information from health care professionals, history of multiple sexual partners, sexually transmitted infection, having high knowledge score and high attitude score were found to be statistically significant. Respondents with age range 30-34 years were two point nine times more likely to practice cervical cancer screening than women whose age range 25-29 years (AOR= 2.987, 95% CI (1.626, 5.49)). Women those who got information from health professionals were two point five times more likely to practice screening than those who did not mention health professionals as source of information (AOR=2.521, 95% CI (1.487,4.275)) shown on Table 2.

**Table 2:** bivariate and multivariate analysis of factors associated with cervical cancer screening practice among women employees in hospitals Wolaita Zone, Ethiopia, June 2017

Variables	Cervical cancer screening		COR, 95%CI	AOR, 95%CI
	Yes	No		
**Age (years)**				
25-29	40	155	1.714(0.731, 4.021)	
30-34	68	83	3.191(1.995, 5.106	2.987(1.626, 5.49)*
35-49	7	21	2.008(0.865, 4.660)	
40-44	4	6	2.891(0.745, 11.224)	
45-49	3	5	1	1
**Occupation**				
Health care professionals	76	96	3.299(1.927, 5.647)	4.103(2.282, 7.377*
Administrative staff	8	63	0.529(0.224,1.25)	
Adherence supporters	12	12	4.167(1.667,10.412)	3.741(1.414, 9.899*
Cleaners	24	100	1	1
**Source of information from health professionals**				
Yes	79	63	2.696(1.734,4.191)	2.521(1.487, 4.275*
No	192	57	1	1
**Source of information from printed materials**				
Yes	10	19	1.908(0.888,4.102)	
No	110	252	1	
**Sex with more than one partner**				
Yes	73	60	2.712(1.734,4.242)	2.289(1.336, 3.922*
No	60	198	1	
**STI**				
Yes	50	44	3.685(2.267,5.99)	3.13(1.784, 5.493)*
No	70	227	1	
**Knowledge**			1.268(1.291,1.542)	1.267(1.192, 1.346*
**Attitude**			1.468(1.374,1.716)	1.468(1.334, 1.616*

Key points; 1= reference, *=p<0.05

## Discussion

Study was conducted to assess cervical cancer screening practice and associated factors among women employees. The study revealed that 120 (30.5%) of age eligible women employees have been screened for cervical cancer. The current cervical cancer-screening practice (30.5%) is higher when compared to study done in Mekele town and in Uganda [7,12]. This might be due to difference in socio-demographic characteristics and screening has become a routine procedure in current study area and working environment exposure and awareness creation interventions such as health education on cervical cancer screening might be high when compared to previous one. On the contrary, this study is lower than study conducted in Botswana. This may partly be due to difference in the socio-demographic characteristics of the study subjects, difference in health delivery system and priority given by two countries and in Botswana Ministry of Health´s had set goal to reach cervical cancer screening for at least 75% and more nationally [8].

From this study it was found that, age of study participant was one of the significant predictors of cervical cancer screening uptake. Women from age 30-34 was 2.9 times more likely to be screened compared to women in age range 25-29 years (AOR = 2.987 (1.626, 5.49) 95% CI. The higher screening rates among this age group are consistent with study done in Mekele and study done in elsewhere in Africa [12,17]. This might be due to information´s and related facts dissemination. Nowadays, information´s about cervical cancer are more probably focused on age group greater than 30 years and WHO recommendations put age range from 30-49 for all women and below that for women at high risk to be screened and most of the individual woman sees her as being at risk and seeks care after recognizes symptoms and perceive susceptibility.

Occupation of study participants was one of the significant variables associated with cervical cancer screening practice. Working as counselor or as adherence supporter in ART was 3.74 times more likely associated with screening practice (AOR=3.741 (1.414, 9.899)) and being health care professionals was 4.103 times more likely associated with screening practice (AOR=4.103 (2.282, 7.377)). This finding is consistent with study done in Jimma town, southwest Ethiopian [18]. This might be because of the majority of employed women have higher educational status working in the hospital and they have access to information about cervical cancer and screening service. Another finding of the present study indicated that history of multiple sexual partners were also important predictor of cervical cancer screening uptake. Women who have admitted having a recent history of multiple sexual partners were 2.289 times more likely to undergo screening compared to those who did not have such history 2.289 (AOR=1.336,3.922). This finding was higher but consistent with study done in Mekele and study done in Africa respectively [8,12]. This might be perception of risk exposure status and educational back ground of the respondent. On the top of that the more sexual partners a woman has, the greater are her chances of becoming infected with human Papilloma virus which is the most common risk factors for development of the cervical cancer.

Another finding related to this was women with sexually transmitted infection was 3.13 times more likely to be screened than those without STI (AOR=3.13 (1.784, 5.493). The same result was also reported from Botswana [19]. Women with frequent use of physician services do not afraid to expose their genitalia and those requesting annual general and gynecological examinations had a higher probability of having had cervical cancer screening and more likely to seek screening for cervical cancer. Source of information from health care professionals were predictor variable and women who heard information from health care professional were 2.521 times more likely to practice cervical cancer screening compared with women who did not hear from health professionals (AOR=2.521 (1.487,4.275)). This was consistent in study done in Gonder [20]. The reason might be health professionals are more acceptable in the community and information heard from them could be loyal and study participants in current study were those health professionals and those working with them.

Knowledge and attitude was another important variable predicts screening practice. As knowledge score increased by one, the cervical cancer screening practice was increased by 1.267 (AOR=1.267 (1.192, 1.346). This finding is in line with study done in Botswana, Mekele and Jimma [12,18,19]. The reason might be due to knowledge about cervical cancer increase their awareness about the advantage of undergoing cervical cancer screening. Those having high score in attitude were practice 1.468 more likely (AOR= 1.468 (1.334, 1.616). This finding is inconsistence with study done in Zambia, Uganda and Botswana [7,8,21]. The reason due to the fact that the awareness of having screening of cervical cancer is now a day is increasing because of the seriousness of the problem and the other reason might be the study area setting in which study participants are working in the health institution. In order to increase the cervical cancer screening practice and preventing the risk of developing cervical cancer in the country, future research is recommended upon cervical cancer screening practice uptake and its barrier for all age eligible women as a national level in Ethiopia

**Limitation of the study:** cross-sectional nature of the study that might be bringing bias. This study is conducted in one zone public hospitals and therefore can´t be generalized to other hospitals in Ethiopia.

## Conclusion

Magnitude of cervical cancer screening service uptake among age eligible women is still low. Age of the women, adherence supporter, and source of information from health care professionals, history of multiple sexual partners, sexually transmitted infection, knowledge and attitude were important predictors of cervical cancer screening practice. Wolaita zonal health department in collaboration with other stake holders should make much efforts to promote cervical cancer screening among women employees and disseminating information that focuses on educating the women about cervical cancer risks and efforts should focus on informing and educating women employees in different institutions. Hospitals in collaboration with town and district administrator should put priority on cervical cancer prevention by establishing cervical cancer screening campaign. In general, health-care authorities do for improving cervical cancer screening by using trained and skilled professionals and distributing adequate supplies, laboratory infrastructure and equipment in the health institution and giving special concern and priority for reproductive age women problems at zonal level in particular and as a whole in the country.

### What is known about this topic

- Knowledge, attitude and practice of cervical cancer screening as well as its service utilization have been widely done in other parts of the world;
- Lifetime prevalence of cervical cancer screening was done in 55 low- and middle-income countries but including Ethiopia.

### What this study adds

- Cervical cancer screening practice is associated with having multiple sexual partners, adherence supporter and with history of sexually transmitted infection;
- Despite few studies on practice of cervical cancer screening were done in Ethiopia, none exist in the study area until this research is conducted;
- Therefore, this study brings in insights into the study area about the advantage of having screening practice in early detection and management of cervical cancer.
